# Genome Sequence of Acetomicrobium hydrogeniformans OS1

**DOI:** 10.1128/genomeA.00581-18

**Published:** 2018-06-28

**Authors:** Lauren E. Cook, Spencer S. Gang, Alicia Ihlan, Matthew Maune, Ralph S. Tanner, Michael J. McInerney, George Weinstock, Elizabeth A. Lobos, Robert P. Gunsalus

**Affiliations:** a Department of Microbiology, Immunology, and Molecular Genetics, University of California, Los Angeles, California, USA; b Department of Microbiology and Plant Biology, University of Oklahoma, Norman, Oklahoma, USA; c The Genome Institute, Washington University School of Medicine, St. Louis, Missouri, USA; d UCLA DOE Institute, University of California, Los Angeles, California, USA

## Abstract

Acetomicrobium hydrogeniformans, an obligate anaerobe of the phylum Synergistetes, was isolated from oil production water. It has the unusual ability to produce almost 4 molecules H_2_/molecule glucose. The draft genome of A. hydrogeniformans OS1 (DSM 22491^T^) is 2,123,925 bp, with 2,068 coding sequences and 60 RNA genes.

## Abstract (precis)

Acetomicrobium hydrogeniformans, an obligate anaerobe of the phylum Synergistetes, was isolated from oil production water. It has the unusual ability to produce almost 4 molecules H_2_/molecule glucose.

## GENOME ANNOUNCEMENT

Acetomicrobium hydrogeniformans strain OS1^T^, until recently known as Anaerobaculum hydrogeniformans (1), of the phylum Synergistetes, is capable of producing almost four molecules of hydrogen per glucose molecule, the theoretical maximum (2, 3). It ferments other substrates, including amino acids, dicarboxylic acids, and other sugars, and can respire using several sulfur compounds. NaCl is an absolute requirement for growth, which suggests a sodium-based energy strategy (2, 4, 5). Although it can reduce elemental sulfur, thiosulfate, and l-cysteine to sulfide, the pathways are unknown. The genome is currently divided among eight scaffolds, one of which contains 2,103,414 bp and seven of which are small scaffolds ranging from 507 bp to 7,345 bp. The genome lacks genes needed for respiration of sulfate and nitrate and genes for *b*- and *c*-type cytochromes. The metabolic strategy appears to be limited primarily to fermentation of simple sugars, amino acids, and certain dicarboxylic acids in addition to an as-yet undescribed respiratory mode for elemental sulfur or thiosulfate. Despite the ability to ferment sugars, the Embden-Meyerhof-Parnas, pentose phosphate, and Entner-Doudoroff pathways appear to be incomplete. It also lacks a complete set of trichloroacetic acid (TCA) genes. OS1^T^ possesses two gene clusters with hydrogenase-related functions to form molecular hydrogen. It also possesses genes for two putative formate dehydrogenases and a formate hydrogenlyase complex. It has multiple genes for stress adaptation, including those for motility, pili, osmotic adaptation, heavy-metal resistance, multidrug export, and toxin/antitoxin synthesis. It has a 21-gene cassette for the synthesis of a carboxysome-like shell structure. Although motility was not previously reported (2), motility can be observed in wet mounts of cultures, and negative-stained cells exhibit polar flagella (R. S. Tanner, personal communication). The genome contains a complete inventory of genes needed to make flagella and multiple genes for chemoreception and chemotaxis. The genome also contains a complement of 10 *pil/pul* genes needed for synthesis and assembly of a type IV pilus apparatus. It has a relatively small number of primary transcription factors (43 genes) and six predicted two-component regulatory systems, including the aforementioned chemotaxis signaling systems. Thus, OS1 appears to have adopted a minimal regulatory strategy, although the substrate range is considerable with respect to the variety of sugars, amino acids, and dicarboxylates used (2). Little is known about translational or posttranslational control operating in this or other Synergistetes species. Strikingly, the genome lacks genes for the prototypical bacterial F_o_F_1_-type ATP synthase but possesses instead three clusters of genes encoding either archaeal-like or vacuolar-like A/V ATP synthases. It also has other archaeal-like annotated genes, including those acting in glycolysis; of note, we found four putative candidates for glyceraldehyde-3-phosphate oxidoreductase, a ferredoxin-utilizing enzyme with archaeal properties. Other genes with archaeal properties include those related to formyl methanofuran dehydrogenase A, B, C, D, and E subunits, methanyltetrahydromethanopterin cyclohydrolase; and Formylmethanofuran-tetrahydromethanopterin *N*-formyltransferase. These genes may have been acquired by lateral gene transfer (6–8).

### Accession number(s).

This whole-genome shotgun project has been deposited in GenBank under the accession no. ACJX00000000.
